# Structural basis of enzyme activity regulation by the propeptide of l-lysine α-oxidase precursor from *Trichoderma viride*

**DOI:** 10.1016/j.yjsbx.2021.100044

**Published:** 2021-01-13

**Authors:** Masaki Kitagawa, Nanako Ito, Yuya Matsumoto, Masaya Saito, Takashi Tamura, Hitoshi Kusakabe, Kenji Inagaki, Katsumi Imada

**Affiliations:** a Department of Macromolecular Science, Graduate School of Science, Osaka University, 1-1 Machikaneyama-cho, Toyonaka, Osaka 560-0043, Japan; b Department of Biofunctional Chemistry, Graduate School of Environmental and Life Science, Okayama University, Okayama 700-8530, Japan; c Enzyme Sensor Co., Ltd., Tsukuba, Ibaraki 305-0047, Japan

**Keywords:** LysOX, l-lysine α-oxidase, LAAO, l-amino acid oxidase, L-Lysine α-oxidase, Crystal structure, Precursor, Substrate recognition

## Abstract

Harmuful proteins are usually synthesized as inactive precursors and are activated by proteolytic processing. l-Amino acid oxidase (LAAO) is a flavoenzyme that catalyzes the oxidative deamination of l-amino acid to produce a 2-oxo acid with ammonia and highly toxic hydrogen peroxide and, therefore, is expressed as a precursor. The LAAO precursor shows significant variation in size and the cleavage pattern for activation. However, the molecular mechanism of how the propeptide suppresses the enzyme activity remains unclear except for deaminating/decarboxylating *Pseudomonas*l-phenylalanine oxidase (PAO), which has a short N-terminal propeptide composed of 14 residues. Here we show the inactivation mechanism of the l-lysine oxidase (LysOX) precursor (prLysOX), which has a long N-terminal propeptide composed of 77 residues, based on the crystal structure at 1.97 Å resolution. The propeptide of prLysOX indirectly changes the active site structure to inhibit the enzyme activity. prLysOX retains weak enzymatic activity with strict specificity for l-lysine and shows raised activity in acidic conditions. The structures of prLysOX crystals that soaked in a solution with various concentrations of l-lysine have revealed that prLysOX can adopt two conformations; one is the inhibitory form, and the other is very similar to mature LysOX. The propeptide region of the latter form is disordered, and l-lysine is bound to the latter form. These results indicate that prLysOX uses a different strategy from PAO to suppress the enzyme activity and suggest that prLysOX can be activated quickly in response to the environmental change without proteolytic processing.

## Highlights

• The suppression mechanism of activity by propeptide remains unclear for most LAAOs.
• The crystal structures of the LysOX precursor (prLysOX) have been determined.
• The propeptide indirectly changes the active site structure to suppress the activity.
• prLysOX can adopt another conformation similar to mature LysOX.
• prLysOX is able to be activated without proteolytic processing.

## Graphical abstract

**Figure:**
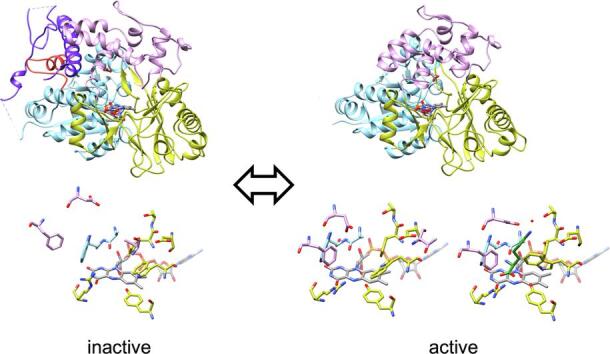

## 1 Introduction

Many toxic or harmful proteins are expressed as inactive precursors and are activated by cleavage of specific sites and removal of the propeptide when their function is required. To prevent an undesirable reaction in the cell, the propeptide suppresses the catalytic activity of the hazardous enzymes. The propeptide also acts as an intramolecular chaperone that assists in proper folding for some enzymes. The role and functional mechanism of propeptide are well studied for proteases (Khan and James, 1998). X-ray structural studies of zymogens and corresponding mature proteases have shown the structural changes induced by the cleavage of the zymogens and revealed the mechanisms of the enzyme activity regulation by the propeptides.

l-Amino acid oxidase (LAAO) is an FAD-dependent enzyme that catalyzes the oxidative deamination of l-amino acid to produce a 2-oxo acid (Pollegioni et al., 2013) and is found in various organisms, such as venomous snakes, mammals, insects, fishes, mollusks, fungi, and bacteria (Yu and Qiao, 2012). Because LAAO produces hydrogen peroxide, which is highly toxic to living organisms, during the LAAO catalysis, most of the LAAOs are expressed as precursor proteins and are processed by proteases for activation. LAAOs of snake venom are synthesized as precursors having 18 extra residues at their N-termini (Ullah, 2020, Takatsuka et al., 2001). LAAO from *Neurospora crassa* synthesized as a precursor composed of 695 amino acids, and its N-terminal 129 residues are cleaved to generate the mature enzyme (566 amino acids) (Niedermann and Lerch, 1990). LAAO from *Rhodococcus opacus*, a gram-positive bacterium, is produced as a precursor with a propeptide of 45 amino acids (Geueke and Hummel, 2002). While these LAAOs are activated by removal of the N-terminal pro-sequence, l-glutamate oxidase from *Streptomyces* sp. X-119–6 (LGOX) needs more processing for activation in addition to the elimination of the N-terminal propeptide (Arima et al., 2009). LGOX is expressed as a single polypeptide precursor composed of 701 residues. The precursor shows weak enzyme activity and is cleaved into three polypeptide chains to generate the mature enzyme. The N-terminal 14 residues, 40 residues between the second, and the third chains and the C-terminal 18 residues are removed in the active mature enzyme.

l-phenylalanine oxidase from *Pseudomonas* sp. P-501 (PAO), a deaminating and decarboxylating LAAO, consists of two polypeptides produced by cleavage of a single polypeptide precursor. The N-terminal 14 residues and residues 107–108 are removed during maturation. The structural study of the precursor and the mature protein has revealed that the N-terminal 14 residues of the propeptide occupy the pathway to the active site thereby inhibit the enzyme activity (Ida et al., 2008). However, the inactivation mechanism by the propeptide remains unclear for all other LAAOs.

l-Lysine oxidase (LysOX) is a member of the LAAO family protein and has strict substrate specificity for l-lysine (Kusakabe et al., 1980). Like other LAAOs, LysOX is produced as an inactive precursor and is processed by proteases for activation. LysOX from *Scomber japonicus*, which induces apoptosis of the fish cell to impede nematode infection, is expressed as a precursor containing an N-terminal propeptide composed of 30 residues (Jung et al., 2000). LysOX from *Aplysia californica*, which is a component of the ink of *A. californica* used as a chemical weapon to escape from predators, has 18 extra residues in its N-terminus in the pro-form (Yang et al., 2005).

In contrast to these LysOX proteins, LysOX from *Trichoderma viride* has much longer propeptide in its N-terminus. LysOX from *Trichoderma viride* is synthesized as a precursor (prLysOX) and the N-terminal 77 residues are cleaved to produce the mature protein composed of 540 amino acid residues (Amano et al., 2015). The mature enzyme consists of three domains; the FAD-binding domain, the helical domain, and the substrate-binding domain (Amano et al., 2015). The active site is present at the bottom of the funnel formed between the substrate-binding domain and the helical domain. A recent structural study has revealed that the triangular arrangement of D289 (The residue numbering includes the 77 residues of the N-terminal pro-sequence in this article) and the two fixed water molecules, one is bound to D392 and the other to the mainchain oxygen of A517, plays the key role for strict recognition of l-lysine as well as the narrow hole formed by the hydrophobic residues (Kondo et al., 2020).

Since the N-terminal propeptide is much longer than that of PAO, proLysOX is expected to use a different way to suppress the enzyme activity than PAO. However, the molecular mechanism of how the propeptide region of LysOX regulates the enzyme activity remains unknown. Here we have determined the crystal structure of prLysOX at 1.97 Å. The structure revealed that the residues involved in the substrate recognition are moved by the structural change of the helical domain, which is induced by the formation of the 8-turn helix composed of the N-terminal half of the propeptide. Thus, the propeptide of prLysOX indirectly changes the active site structure to suppress the enzyme activity. prLysOX shows weak enzymatic activity with strict specificity for l-lysine and raised activity in acidic conditions. The structures of prLysOX crystals soaked in a solution with various concentrations of l-lysine show that prLysOX can adopt two conformations, one of which is very similar to mature LysOX. These results indicate that prLysOX uses a different strategy from PAO to suppress the enzyme activity and suggest that prLysOX can be activated in response to the environmental change without proteolytic processing.

## 2 Results

### 2.1 Enzymatic property of the LysOX precursor

**Fig. 1:**
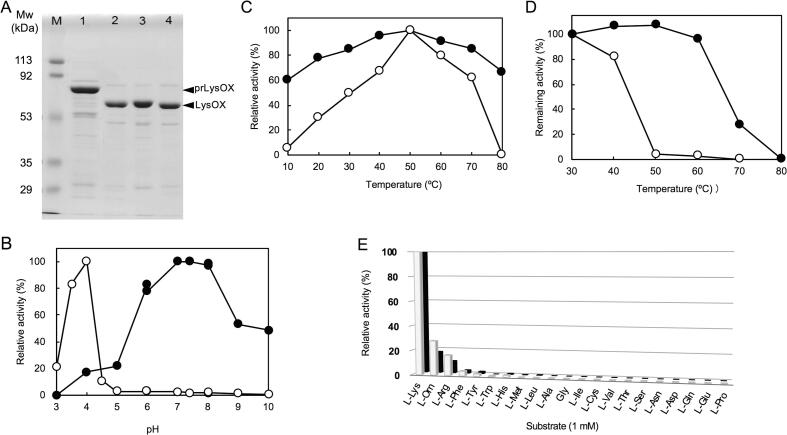
Comparison of enzyme properties of prLysOX with mature LysOX. (A) Cleavage of the propeptide region by various proteases. The proteolysis products of prLysOX were examined by SDS-PAGE: M, molecular weight marker; lane 1, prLysOX; lane 2, after treatment with trypsin; lane 3 after treatment with chymotrypsin; lane 4, after treatment with SGMP. (B) Effect of pH on the enzyme activities of prLysOX and mature LysOX. Relative activities of prLysOX (open circle) and mature LysOX at 40 °C (closed circle) under various pHs are plotted. The activity at optimum pH (pH 4.0 for prLysOX and pH 7.4 for mature LysOX) was set as 100% relative activity. The buffers used for the enzymatic assay are as follows: citrate buffer (pH 3.0–6.0), KPB (pH 6.0–8.0), sodium borate buffer (pH 8.0–10.0). (C) Temperature dependence of the enzyme activities of prLysOX and mature LysOX. Relative oxidative activities of prLysOX at pH 4.0 (open circle) and mature LysOX at pH 7.4 (closed circle) under various temperatures are plotted. The activity at 50 °C was set as 100% relative activity. (D) The remaining activities after incubation for 30 min at various temperatures. Relative oxidative activities of prLysOX at pH 4.0 (open circle) and mature LysOX at pH 7.4 (closed circle) are plotted. The oxidative activity without heat treatment was set as 100% remaining activity. (E) Substrate specificity of prLysOX and mature LysOX. Relative oxidative activities of prLysOX and mature LysOX are shown in gray and black bars, respectively. The specific activity against l-lysine (prLysOX: 2.0 U/mg, mature LysOX: 54.5 U/mg) was set as 100% relative activity. Enzyme activities were measured with 1 mM substrates at 40℃ and pH 7.4.

prLysOX overexpressed in *E. coli* was purified as previously described (Kondo et al., 2020). prLysOX showed a low specific activity of less than 1/50 of mature LysOX, suggesting that removal of the propeptide region activates LysOX. To clarify whether the removal of the propeptide increases the enzyme activity, we examined various proteases, such as trypsin, chymotrypsin, and metalloendopeptidase from *Streptomayces griseus* (SGMP), to cleave off the propeptide region of prLysOX and assessed enzyme activity of the proteolytic products. prLysOX is digested to produce a stable fragment of 56 kDa, which is comparable to the molecular mass of native LysOX (Fig. 1A). We analyzed the N-terminal sequence of the fragments and found that all of them begin with AE, which is the same as the N-terminal sequence of native LysOX purified from *T. viride* (Amano et al., 2015). After proteolysis, LysOX exhibited the specific activity of 66.1 U/mg (Kondo et al., 2020), which is almost the same value of specific activity of LysOX purified from *T. viride* (66.2 U/mg (Kusakabe et al., 1980)). Therefore, we concluded that the propeptide region suppresses the enzyme activity of LysOX.

Since prLysOX still retains the enzyme activity, we determined the optimum pH and temperature of prLysOX (Fig. 1B and C, Table 1). These values are markedly different from those of the mature protein. The enzyme activity of prLysOX for l-lysine at 40 °C showed its maximum at pH 4.0 (5.9 U mg^−1^) and fell to 3% of the maximum value at pH 5.0. The activity at pH 4.0 was about thirty-fold higher than that at pH 7.0. In contrast, the optimum pH of mature LysOX at 40 °C was pH 7.4 (56.1 U mg^−1^). The activity of mature LysOX was gradually decreased with decreasing pH and was dropped almost 20% of its maximum at pH 4.0. The optimum temperature of prLysOX at pH 4.0 was 50 °C (8.0 U mg^−1^), and that of mature LysOX at pH 7.4 was also 50 °C (56.5 U mg^−1^) (Fig. 1C). The enzyme activity of prLysOX dropped to 80% after 30 min incubation at 40 °C and was lost at 50 °C, whereas that of mature LysOX still retains almost 100% of the activity after 30 min incubation at 60 °C (Fig. 1D). Thus, removal of the propeptide region increases the thermal stability of LysOX.

**Table 1:** Kinetic parameters of prLysOX and mature LysOX.

	*K*_m_ (mM)	*k*_cat_ (s^−1^)	*k*_cat_/*K*_m_ (s^−1^ mM^−1^)
prLysOX	0.28	6.1	22
mature LysOX	1.3 × 10^−2^	65.5	5.0 × 10^3^

The kinetic parameters of prLysOX were measured at 50 °C, pH 4.0, and those of mature LysOX at 50 °C, pH 7.4.

Next, we examined the substrate specificity of the precursor. prLysOX exhibits a similar substrate specificity profile to LysOX (Fig. 1E). l-ornithine, l-arginine, l-phenylalanine, l-tyrosine, and l-histidine showed weak activity, but no activity was detected for other amino acids. Therefore, prLysOX retains strict specificity for l-lysine.

**Fig. 2:**
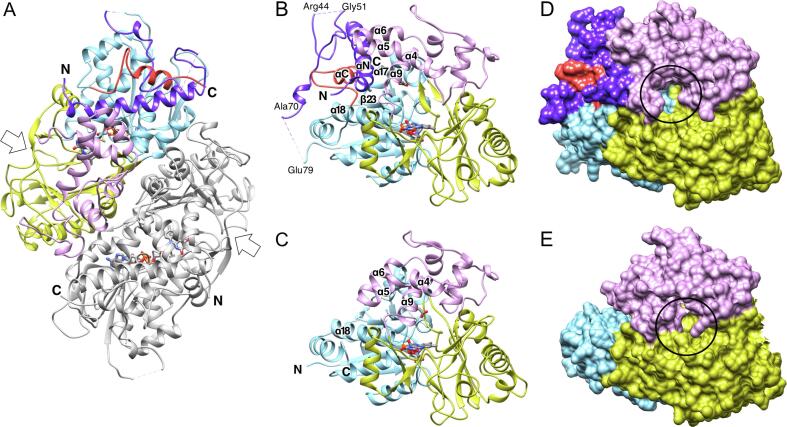
Structure of prLysOX. (A) Ribbon representation of the prLysOX dimer. One subunit is colored by domain: cyan, the FAD-binding domain; yellow, the substrate-binding domain; magenta, the helical domain; purple, the propeptide region; red, the C-terminal region. Its dimer mate related by crystallographic two-fold symmetry is colored in gray. (B) (C) Ribbon diagrams of a single subunit of prLysOX (B) and mature LysOX (C) are viewed from the funnel entrance. (D) (E) Surface representations of a single subunit of prLysOX (D) and mature LysOX (E) viewed from the same directions as (B) and (C), respectively. The domains are painted in the same color codes as (A). FAD is shown by stick colored by element: red, oxygen; blue, nitrogen; grey, carbon; orange, phosphorus. The non-visible loops are shown by broken lines. The entrance of the funnel is shown by white arrow in (A) and black circle in (D) and (E). (For interpretation of the references to color in this figure legend, the reader is referred to the web version of this article.)

**Structure of the LysOX precursor.** The crystal structure of prLysOX was determined at 1.97 Å resolution (Fig. 2). A single prLysOX molecule is in an asymmetric unit and forms a dimer with the neighboring molecule related by crystallographic two-fold symmetry. prLysOX consists of 617 amino acid residues, and the final model contains N3–R44, G51–A70, and E79–K611.

The propeptide (M1–R77) of LysOX folds into a sub-domain on the FAD-binding domain with the C-terminal region (Q590–K611), which is disordered in the mature LysOX structure (Fig. 2). The propeptide region consists of an 8-turn amphiphilic α-helix (F6–L34, αN), a proline/glycine-rich region that forms a repeat of turn structures (G35–P62), and a loop with a short helical segment (L63–R72). The N-terminal two residues (M1–D2), the six residues (E45–G50) in the proline/glycine-rich region, and the C-terminal eight residues of the propeptide leading to the N-terminus of mature LysOX (L71–A78) were not included in the final model because of poor electron density. The C-terminal 28 residues (Q590–I617), which were non-visible in the LysOX structure, appeared in the prLysOX structure except for the C-terminal six residues (E612–I617). The residues A600–G607 form an α-helix (αC) located in the cleft formed by the C-terminal half of αN, α18, and the loop connecting α17 with β23.

**Fig. 3:**
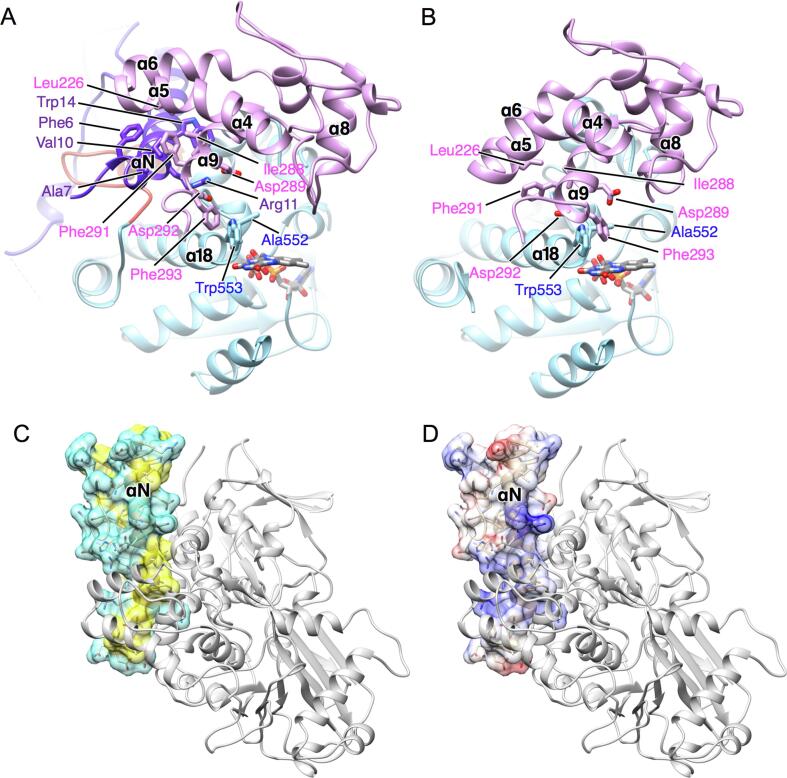
The structural difference between prLysOX and LysOX. (A) (B) Ribbon representation of prLysOX (A) and LysOX (B). The substrate-binding domain is removed for clarity. The domains and FAD are colored in the same color as Fig. 2. The residues involved in substrate binding and in the interaction between αN and the helical domain are indicated by stick. (C) (D) Interaction surface of the propeptide with the helical and the substrate-binding domains shown by gray ribbon. (C) Amphiphilic feature of the interface. Polar and hydrophobic residues are colored in cyan, and non-polar residues in yellow. (D) The electrostatic property of the interface. (For interpretation of the references to color in this figure legend, the reader is referred to the web version of this article.)

Unlike the precursor of PAO (Ida et al., 2008), the propeptide is far from the entrance of the funnel leading to the active site (Fig. 2B and D). The propeptide induces a structural change in the helical domain, but no significant change is found in the other two domains (Figs. 2B, C, and 3). αN is inserted between the helical domain and the FAD-binding domain. The N-terminal region of αN pushes up α5 and α6 resulted in straightening the kink between α4 and α5 and induces a movement of α9 to form a tight hydrophobic interaction. I228 in α9 directly interacts with L226 in α5 in LysOX. However, I288 forms hydrophobic interactions with V10, W14, and aliphatic side-chain arm of R11 in αN in prLysOX. F291, which is in α9 and interacts with α5 and α6 in LysOX, is surrounded by hydrophobic residues of F6, A7, and V10 in αN in prLysOX. In addition to the hydrophobic interaction, a hydrophilic interaction is observed between αN and α9. D289 and D292 in α9 make a charge interaction with R11 in αN.

**Fig. 4:**
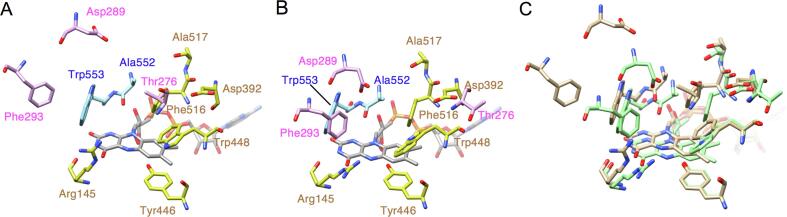
Comparison of the substrate-binding site structure of prLysOX with that of mature LysOX. (A) (B) Substrate-binding site structures of prLysOX (A), and mature LysOX (B). FAD and the carbon atoms of the protein residues are colored with the same color as Fig. 2 (B) and (C). (C) Superimposition of the substrate-binding site structure of prLysOX on that of mature LysOX. The carbon atoms of prLysOX and mature LysOX are shown in light brown and light green, respectively. (For interpretation of the references to color in this figure legend, the reader is referred to the web version of this article.)

The movement of α9 changes the shape of the active site (Fig. 4). The substrate-binding site of LysOX lies at the bottom of the funnel formed between the substrate-binding domain and the helical domain (Fig. 2). The amino acid backbone of l-lysine interacts with R145, Y446, A552, and W553. The sidechain of l-lysine is recognized by a hydrophobic hole formed by F293 W448 F516, A552, and W553 and hydrophilic interactions with D289 and two water molecules bound to D392 and the carbonyl oxygen of A517. D289 and F293 in α9 move away from the active site. T276 in the loop connecting α8 and α9 moves to space where the water mediating the interaction between the ε-amino group of l-lysine and D392 existed. Moreover, the shift of α9 accompanies the movement of the loop in which A552 and W553 are present. These structural changes collapse the active site structure and thus decrease the specific activity of prLysOX for l-lysine.

**Fig. 5:**
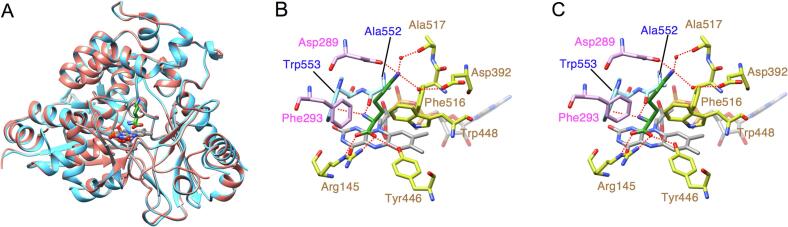
Structural comparison of prLysOX soaked in 1.24 M l-lysine solution and LysOX-Lys. (A) Superimposition of the structure of prLysOX soaked in 1.24 M l-lysine solution (orange) on that of LysOX-Lys (light blue). FAD (gray) and the substrate l-lysine (green) are indicated by stick. (B) (C) Close up view of the substrate-binding site of prLysOX-Lys (B) and LysOX-Lys (C). The substrate l-lysine is colored with the same color as (A). The protein residues and FAD are shown in the same color as Fig. 4 (A). The water molecules involved in the substrate recognition are shown in red balls. The red dot lines indicate the possible hydrogen bonding network and the cation pi interaction involved in substrate recognition. (For interpretation of the references to color in this figure legend, the reader is referred to the web version of this article.)

**Fig. 6:**
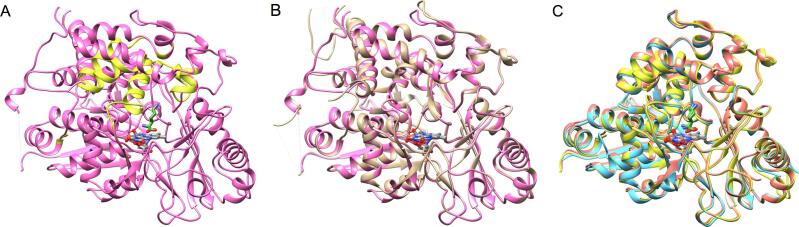
Two conformations in the crystal structure of the prLysOX soaked in 1 M l-lysine solution. (A) Ribbon representation of the crystal structure of prLysOX soaked in 1 M l-lysine solution. The conformation similar to ligand-free prLysOX (conformation-1) is colored in magenta and the other conformation similar to LysOX-Lys (conformation-2) in yellow. (B) Superimposition of the structures of conformation-1 (magenta) and prLysOX (light brown). (C) Superimposition of the structures of conformation-2 (yellow), prLysOX-Lys (orange), and LysOX-Lys (light blue). (For interpretation of the references to color in this figure legend, the reader is referred to the web version of this article.)

**Structure of prLysOX in complex with l-lysine.** The structure of prLysOX revealed that the shift of α9 expands the space of the substrate-binding pocket (Fig. 3, Fig. 4). This structural change is likely to increase the binding affinity for amino acids with bulky side chains while reduces the affinity for l-lysine. However, prLysOX shows a similar substrate specificity profile to LysOX (Fig. 1E). How does prLysOX retain strict specificity for l-lysine? To solve this mystery, we determined the structure of prLysOX in complex with l-lysine. The prLysOX crystals were soaked in reservoir solution containing l-lysine. Unlike LysOX crystals (Kondo et al., 2020), the prLysOX crystals were stable in the soaking solution. The crystals soaked in 50 mM l-lysine solution did not show any change in color, and the structure of the crystal is the same as the prLysOX structure. No electron density corresponding to l-lysine was observed in the active site. In contrast, the crystals soaked in 1 M or 1.24 M l-lysine solution changed color from yellow to colorless, suggesting that FAD is converted to the reduced state and that the substrate is bound to the enzyme (Moustafa et al., 2006, Faust et al., 2007). The structure of the crystal soaked in 1.24 M l-lysine solution was solved at 2.29 Å resolution. Surprisingly, the structure is the same as that of LysOX-Lys (Kondo et al., 2020) (Fig. 5). The propeptide region and the C-terminal helix is disordered. The root mean square deviation (RMSD) for corresponding 497 Cα atoms is 0.31 Å. l-lysine is bound in the same manner as LysOX-Lys (Fig. 5), although the density of l-lysine is not as clear as LysOX-Lys. The structure of the crystal soaked in 1 M l-lysine solution was solved at 1.85 Å resolution. prLysOX in the crystal shows an interesting structure that is a mixture of the structures of prLysOX and the LysOX-Lys with the occupancy ratio of 0.57:0.43 (Fig. 6). Very weak but significant density corresponding to l-lysine was observed in the active site. These results suggest that prLysOX can adopt two conformations and l-lysine binds only to the LysOX like structure. Thereby prLysOX retains strict specificity for l-lysine.

## 3 Discussion

Propeptides of enzyme precursors often brock their active sites or the entrances to the active sites from substrate binding, and thereby inhibit the enzyme activity. The propeptides of many known proteases block their active sites, and processing of the propeptide regions induce structural change that allow the access of the substrates to the active sites of the proteases (Khan and James, 1998). The propeptide of PAO is in the channel to the active site like a plug (Ida et al., 2008). prLysOX, however, uses a different strategy to suppress the enzyme activity. The structure of prLysOX has revealed that the propeptide region of prLysOX does not cover the entrance nor the tunnel to the active site. In addition, the propeptide region does not directly interact with the residues involved in substrate binding or reaction. The propeptide region of prLysOX forms a positively-charged amphiphilic helix, which is inserted between the helical domain and the FAD-binding domain, and changes the structure of the helical domain (Fig. 2, Fig. 3). The structural change moves the active site residues, D289, F293, A552, and W553, which are important for reaction and the substrate binding. Moreover, the structural change shits T276 resulting in the removal of the water mediating the interaction between l-lysine and D392. As a result, the propeptide region of prLysOX indirectly changes the active site structure to inhibit the enzyme activity. Conversely, removal of the propeptide region by proteolysis induces the conformational change of the helical domain followed by the movement of the active site residues to the proper positions to activate the enzyme (Fig. 4).

Some enzymes, such as subtilisin and carboxypeptidase Y, require a propeptide region to form a functional structure (Zhu et al., 1989, Winther and Sorensen, 1991). The N-terminal propeptides of these enzymes assist in the proper folding of following polypeptides. We have ever tried recombinant expression of mature LysOX without the propeptide region in *Streptomyces* or *E. coli* but failed. However, prLysOX was successfully expressed in *Streptomyces* (Amano et al., 2015) or in *E. coli* (Kondo et al., 2020), suggesting that the propeptide region is essential for LysOX production. The N-terminal amphiphilic helix in the propeptide region interacts with the FAD-binding domain and the helix domain as well as the C-terminal region of LysOX. Thus, it would be possible that the propeptide region of LysOX acts as an intramolecular chaperone and assists in the proper folding of the enzyme.

prLysOX transformed into a similar structure to LysOX under extremely high substrate concentration. The substrate complex of prLysOX was prepared by the soaking method, and the structural change occurred without changing the molecular packing in the crystal. Interestingly, the structural transition depends on substrate concentration. A mixed structure of the propeptide-folded form and the propeptide-disordered form was observed at slightly low substrate concentration, and no structural change occurred at low substrate concentration. These observations suggest that structural change is not a result of unexpected cleavage of the propeptide by contaminated proteases during crystallization, but is caused by the addition of the substrate. It is unclear how the extremely high substrate concentration induces the structural change of the propeptide region as well as the active site. Under extremely high l-lysine concentration, l-lysine can weakly bind to prLysOX through its amino acid backbone and may stay there long. The thermal fluctuation of the enzyme may occasionally cause a structural distortion that allows interaction between the weakly bound l-lysine and the residues important for the substrate binding, and such interactions may trigger the structural change from the propeptide-folded form to the propeptide-disordered form.

The pH profile of the enzyme activity is different between prLysOX and mature LysOX, although the substrate binding structure of prLysOX resembles that of LysOX. The enzyme activity of prLysOX sharply increased around pH 4.0 and reaches the maximum value at pH 4.0, whereas that of LysOX shows its maximum around pH 8.0 and gradually drops with a decrease of the pH value (Fig. 1B). The side chain of l-lysine is recognized by two aspartate residues, and the surface around the substrate-binding site is negatively charged by acidic residues (Kondo et al., 2020). The side chain pKa of glutamate and aspartate is around 4.0, and thus acidic environment is likely to be unsuitable for the l-lysine binding to LysOX. In fact, the relative activity of mature LysOX at pH 4.0 is 20% of its maximum, and the Km value of prLysOX at pH 4.0 is 20 times larger than that of LysOX at pH 7.4. Then why prLysOX shows the maximum activity at pH 4.0? The sharp and large change of the enzyme activity implies a structural change of prLysOX. The calculated isoelectric point of the propeptide region is 9.29, whereas that of the mature LysOX is 5.47. The propetptide region contains 13 positively charged residues (nine arginine and four lysine residues) and 11 negatively charged residues (4 aspartic acid and 7 glutamic acid residues). Thus, the propetptide region is positively charged (Fig. 3D). The positively charged surface interacts with the acidic surface of LysOX. The positively charged residues in αN, such as R11, R17, and R20, interact with the acidic residues, such as D231, D235, D289, D292, and E593. These interactions contribute to stabilize the propetptide structure. Because the side chains of glutamate and aspartate residues are neutralized under acidic condition, these interactions will be lost around pH 4.0, leading to destabilize the propeptide structure. As a result, the propeptide region may be disordered around pH 4.0, and the prLysOX structure may be changed into a similar structure to LysOX. Thus, prLysOX can be activated quickly in response to the environmental change, for example, acidification, without proteolytic processing.

## 4 Methods

**Expression and purification of prLysOX.** prLysOX is expressed in *Escherichia coli* SoluBL21 as previously described (Kondo et al., 2020). The cells were harvested by centrifugation, suspended in 20 mM potassium phosphate buffer (KPB) at pH 7.4 containing 1 mM ethylenediaminetetraacetic acid (EDTA) and phenylmethylsulfonyl fluoride (PMSF), and disrupted by sonication on ice. After removal of cell debris by centrifugation at 10,000×*g* for 20 min at 4 °C, ammonium sulfate was added to the supernatant to 65% saturation, and the solution was stored at 4 °C for 30 min. The precipitate was collected by centrifugation at 10,000×*g* for 30 min at 4 °C, dissolved, and dialyzed against 20 mM KPB at pH 7.4 containing ammonium sulfate at 20% saturation. The solution was loaded on a Butyl-Toyopearl 650 M column (Tosoh) equilibrated with 20 mM KPB at pH 7.4 containing ammonium sulfate at 20%. The bound enzyme was eluted with 20 mM KPB at pH 7.4 containing 10% saturated ammonium sulfate. The enzymatically active fractions were concentrated using Amicon Ultra centrifugal filters with a 30 kDa cut-off (Merck Millipore, USA), and dialyzed against 20 mM KPB at pH 7.4. The solution was applied to a DEAE–Toyopearl 650 M column (Tosoh) equilibrated with 20 mM KPB at pH 7.4. The bound enzyme was eluted with 20 mM KPB at pH 7.4 containing 200 mM NaCl. The enzymatically active fractions were concentrated by Amicon Ultra centrifugal filters with a 30 kDa cut-off. The purity of the enzyme was examined by sodium dodecyl sulfate–polyacrylamide gel electrophoresis (SDS–PAGE). All purification procedures were performed at 0–4 °C.

**Proteolysis of the N-terminal propeptide.** 2 mg·ml^−1^ of purified prLysOX was mixed with 1 mg·ml^−1^ of each protease with a volume ratio of 50:1, and the reaction mixture was incubated at 37 °C for 4 h. The reaction was stopped by adding EDTA and PMSF to final concentrations of 1 mM each.

**Determination of N-terminal amino acid sequence.** The purified enzymes were blotted onto a polyvinylidene difluoride (PVDF) membrane after SDS-PAGE. The PVDF membrane was stained with Coomassie brilliant blue. The protein band was excised from the membrane and used to determine the N-terminal amino acid sequence by Edman degradation using PPSQ-31A protein sequencer (Shimadzu).

**Enzyme assay.** The enzyme activity was measured by detecting hydrogen peroxide using the color development method with 4-aminoantipyrine, phenol, and horseradish peroxidase (Job et al., 2002). One unit of enzyme activity was defined as the amount of enzyme catalyzing the production of 1 μmol of hydrogen peroxide per minute. The standard assay mixture contained 70 mM KPB at pH 7.4, 5 mM l-lysine for prLysOX, 1 mM l-lysine for mature LysOX, 10 U ml^−1^ horseradish peroxidase, 0.5 mM 4-aminoantipyrine, 1.7 mM phenol, and an appropriate amount of enzyme in a final volume of 1.0 ml. The reaction was initiated by the addition of enzyme solution and was monitored by absorbance at 505 nm at 40℃ for 3 min using a spectrophotometer UV mini 1240 (Shimadzu, Japan). Protein concentration was determined by Protein assay kit (Bio-Rad) using BSA as a standard. The apparent kinetic parameters *k*_cat_ and *K*_m_ were calculated from the reaction rates determined at various concentrations of substrate l-amino acids using the Lineweaver-Burk plot.

**Crystallization, data collection, and structure determination of prLysOX and its complex with l-lysine.** Crystallization was carried out using the sitting-drop vapor-diffusion method. Crystallization drops were prepared by mixing the prLyOX solution with an equal volume of reservoir solution. Initial screening was carried out using screening kits (Cryo I and II (Emerald Biostructures) and Crystal Screen I and II (Hampton Research)), and then the conditions were optimized. The crystals used for X-ray data collection were grown at 20 °C from drops prepared by mixing 0.5 μL protein solution (5 mg mL^−1^) containing 20 mM potassium phosphate at pH 7.4 with the equivalent volume of reservoir solution containing 0.1 M Tris-HCl (pH 8.0 – 9.0) and 2 – 2.1 M ammonium dihydrogen phosphate. The crystals belong to the space group *C*222_1_ with unit cell dimensions of *a* = 95.2, *b* = 130.9, and *c* = 94.1 Å. The prLysOX-Lys crystals were prepared by the soaking method. The prLysOX crystals were transferred into a reservoir solution containing 1.24 M or 1.0 M l-lysine and were stored at 20 °C for 80 min.

X-ray diffraction data were collected at Spring-8 (Harima, Japan) beamline BL41XU (Proposal No.2017A2585). Crystals were frozen in liquid nitrogen and mounted in nitrogen gas flow at 100 K. The diffraction data were processed with MOSFLM (Battye et al., 2011) and were scaled with AIMLESS (Evans and Murshudov, 2013). The statistics of the diffraction data are summarized in Table 2. The structures were determined by the molecular replacement method using the structure of LysOX (PDB ID: 3X0V) as a search model with Phaser (McCoy et al., 2007). The atomic models were built with Coot (Emsley et al., 2010) and refined to 1.97 Å, 2.29 Å, and 1.85 Å resolution for prLysOX, prLysOX with 1.24 M l-lysine, and prLysOX with 1.0 M l-lysine, respectively, with PHENIX (Adams et al., 2010). The refinement statistics are summarized in Table 2.

**Table 2:** Data collection and refinement statistics.

	prLysOX	prLysOX-Lys 1.24 M	prLysOX-Lys 1 M
**Data collection**			
Space group	*C*222_1_	*C*222_1_	*C*222_1_
Cell dimensions			
*a*, *b*, *c* (Å)	95.2, 130.9, 94.1	94.3, 128.0, 94.4	94.9, 130.1, 94.3
α, β, γ (°)	90, 90, 90	90, 90, 90	90, 90, 90
Resolution (Å)	94.1–1.97 (2.02–1.97)*	94.4–2.29 (2.37–2.29)	76.7–1.85 (1.89–1.85)
*R*_merge_	0.106 (0.527)	0.074 (0.391)	0.048 (0.322)
*I* / σ*I*	10.3 (2.9)	12.9 (3.2)	16.9 (4.0)
Completeness (%)	93.5 (100.0)	99.4 (99.6)	99.3 (99.0)
Redundancy	5.0 (5.2)	4.1 (4.0)	4.4 (4.5)

**Refinement**			
Resolution (Å)	77.0–1.97 (2.02–1.90)	75.2–2.29 (2.38–2.29)	65.0–1.85 (1.89–1.85)
No. reflections	38,412 (2,906)	25,885 (2,852)	49,596 (2,720)
*R*_work_ / *R*_free_	20.3/23.5 (23.0/26.9)	16.9/22.1 (22.4/28.7)	16.5/20.1 (21.3/30.2)
No. atoms			
Protein	4732	4024	5112
Ligand/ion	68	193	63
Water	570	193	429
*B*-factors			
Protein	23.4	37.9	26.8
Ligand/ion	19.2	27.5	19.1
Water	29.6	37.0	36.1
R.m.s. deviations			
Bond lengths (Å)	0.003	0.008	0.019
Bond angles (°)	0.821	0.925	1.631
Ramachandran plot (%)			
preferred regions	95.6	96.8	95.8
allowed regions	3.9	3.2	4.0
Outliers	0.5	0	0.2

*Values in parentheses are for highest-resolution shell.

## 5 Accession numbers

The atomic coordinates have been deposited in Protein Data Bank, www.pdb.org (PDB ID code 7D4C, 7D4D and 7D4E).

## Author contributions

K Imada and K Inagaki designed research; M.K., N.I., Y.M., M.S., T.T., and H.K. performed experiments, M.K., N.I. and K Imada. analyzed the crystal structure; and K Imada and K Inagaki wrote the paper.

## CRediT authorship contribution statement

**Masaki Kitagawa:** Investigation, Visualization. **Nanako Ito:** Investigation, Visualization. **Yuya Matsumoto:** Investigation, Visualization. **Masaya Saito:** Investigation. **Takashi Tamura:** Investigation. **Hitoshi Kusakabe:** Investigation, Resources. **Kenji Inagaki:** Conceptualization, Funding acquisition, Supervision, Writing - original draft. **Katsumi Imada:** Conceptualization, Project administration, Supervision, Validation, Visualization.

## Declaration of Competing Interest

The authors declare that they have no known competing financial interests or personal relationships that could have appeared to influence the work reported in this paper.
